# Predictors of Early Postoperative Seizures Following STA–MCA Bypass in Adult Moyamoya Angiopathy

**DOI:** 10.3390/jcm15218448

**Published:** 2026-10-30

**Authors:** Riccardo Ciocca, Nazeer Aboud, Erica Boccardi, Daniel Sarmiento, Ricardo Borda, Alessandro Tozzi, Elisa Ciceri, The MoMo-Besta-Team, Marco Paolo Schiariti, Francesco Restelli, Paolo Ferroli, Sergio A. Calero Martinez

**Affiliations:** 1 Department of Neurosurgery, Section of Vascular and Skull Base, Fondazione IRCCS Istituto Neurologico Carlo Besta, Via Celoria 11, 20133 Milan, Italy; riccardo.ciocca@unimi.it (R.C.); erica.boccardi@unimi.it (E.B.); neurochirurgia@istituto-besta.it (T.M.-B.-T.); marco.schiariti@istituto-besta.it (M.P.S.); paolo.ferroli@istituto-besta.it (P.F.);; 2 Department of Neurosurgery, Jena University Hospital, Am Klinikum 1, 07747 Jena, Germany; nazeer.aboud@med.uni-jena.de; 3 Department of Statistics, El Bosque University, Carrera 9 No. 131 A–02, Bogotá 110121, Colombia; dsarmientosar@unbosque.edu.co (D.S.); bordaricardo@unbosque.edu.co (R.B.); 4 SC Radiologia Diagnostica per Immagini e Neuroradiologia Interventistica, Fondazione IRCCS Istituto Neurologico Carlo Besta, Via Celoria 11, 20133 Milan, Italy; alessandro.tozzi@unimi.it (A.T.); elisa.ciceri@istituto-besta.it (E.C.); 5 Department of Neurology, Fondazione IRCCS Istituto Neurologico Carlo Besta, Via Celoria 11, 20133 Milan, Italy; 6 Department for Anesthesiology and Intensive Care Medicine, IRCCS Istituto Neurologico Carlo Besta, Via Celoria 11, 20133 Milan, Italy; 7 Instituto de Neurociencias, El Bosque University, Carrera 9 No. 131 A–02, Bogotá 110121, Colombia

**Keywords:** Moyamoya angiopathy, hyperperfusion, seizures, nova MRI, firth logistic regression

## Abstract

**Background:** Postoperative epileptic seizures are a recognized complication after superficial temporal artery–middle cerebral artery (STA–MCA) bypass for Moyamoya angiopathy (MMA), but their determinants remain incompletely understood. We aimed to identify clinical, radiological, and perioperative factors associated with early postoperative seizures following adult STA–MCA bypass. **Methods:** We retrospectively analyzed 75 hemispheric revascularization procedures performed in adult MMA patients between 2005 and 2023, with each operated hemisphere treated as an independent analytical unit. The primary outcome was the occurrence of at least one in-hospital postoperative seizure; the primary exposure was cerebral hyperperfusion syndrome. Univariate and multivariable Firth penalized-likelihood logistic regression was used to account for the small sample size and rare outcome. **Results:** Postoperative seizures occurred in 14 of 75 procedures (18.7%). Hyperperfusion was identified in 6 procedures (8.0%) and showed the strongest univariate association with seizures (OR 10.20), followed by diabetes mellitus (OR 6.05). Right-hemispheric procedures showed a protective effect in the univariate analysis (OR 0.24). In separate multivariate adjusted models, hyperperfusion (OR 10.28) and diabetes mellitus (OR 5.70) remained associated with postoperative seizures (OR 10.28 and OR 5.70, respectively). Pre-existing epilepsy, antiepileptic treatment, and preoperative EEG abnormalities were not associated with seizure risk. **Conclusions:** Early postoperative seizures after direct STA–MCA bypass in adult MMA were most consistently associated with postoperative cerebral hyperperfusion in our cohort, while pre-existing epilepsy and preoperative EEG abnormalities showed no statistically significant associations. Early recognition of hyperperfusion may support risk-stratified postoperative surveillance and individualized management.

## 1. Introduction

Moyamoya angiopathy (MMA) is a chronic progressive cerebrovascular disorder characterized by stenosis or occlusion of the terminal internal carotid arteries and their proximal branches, resulting in the formation of fragile basal collateral vessels [1,2]. The disease commonly presents with ischemic stroke or intracranial hemorrhage, transient ischemic attacks, cognitive impairment, headaches, and epilepsy, all of which contribute substantially to neurological disability [3,4]. Surgical revascularization is the treatment of choice for symptomatic patients, and direct superficial temporal artery to middle cerebral artery (STA–MCA) bypass provides immediate augmentation of cerebral blood flow while reducing the risk of future ischemic events [5,6].

Although direct bypass is an effective procedure, postoperative neurological complications remain an important cause of morbidity [7]. Among these, postoperative epileptic seizures are of clinical relevance because they may prolong hospitalization, complicate postoperative neurological assessment, increase the need for antiepileptic therapy, and negatively affect functional recovery [8].

Cerebral hyperperfusion syndrome represents one of the best-recognized complications after direct bypass in adult Moyamoya patients and is thought to result from impaired cerebrovascular autoregulation in chronically hypoperfused brain tissue [9]. Excessive regional cerebral blood flow may induce vasogenic edema, oxidative stress, and neuronal hyperexcitability, thereby increasing the risk of postoperative seizures [10]. Some studies have specifically investigated early postoperative seizures following revascularization for MMA, and the reported risk factors have been inconsistent across published series [8,11,12]. Whether postoperative seizures are primarily driven by hyperperfusion or are more closely associated with pre-existing epilepsy, electroencephalographic abnormalities, vascular comorbidities, or baseline clinical characteristics remains incompletely understood.

Therefore, the aim of this study was to identify clinical, radiological, and perioperative factors associated with early postoperative seizures following adult STA–MCA bypass for Moyamoya angiopathy.

## 2. Methods

### 2.1. Study Design and Population

A retrospective cohort study was conducted including all adult patients (≥18 years) with Moyamoya angiopathy who underwent direct or combined direct–indirect surgical revascularization by superficial temporal artery–middle cerebral artery (STA–MCA) bypass at our institution between 2005 and 2023. Patients younger than 18 years at the time of bypass surgery, or incomplete records, as well as those treated exclusively with indirect revascularization, occipital artery–middle cerebral artery (OA–MCA) bypass, or double-barrel bypass procedures, were excluded. A total of 51 patient records were screened. Among these, 24 patients underwent bilateral treatment, and 7 patients underwent direct bypass only. In the cohort, two patients experienced seizures after both the first and second surgical procedures. Each operated hemisphere was considered an independent analytical unit, reflecting the bilateral nature of Moyamoya angiopathy and the procedure-specific risk associated with each revascularization. Overall, 75 hemispheric procedures were included in the primary analysis. Because some patients underwent more than one revascularization procedure, each operated hemisphere was retained as the primary analytical unit to evaluate procedure-specific exposures and outcomes. To assess the potential effect of within-patient correlation, additional sensitivity analyses accounting for patient-level clustering were performed.

### 2.2. Perioperative Management Protocol

Postoperatively, patients were routinely monitored neurologically overnight in the intensive care unit, with strict blood pressure control as the main priority. Systolic blood pressure was maintained at 120–140 mmHg, also taking the patient’s baseline blood pressure into account. Patients were then transferred to the neurosurgical ward on the first postoperative day, where blood pressure was continuously monitored and kept within the same systolic range. Fluid management aimed at euvolemia with isotonic crystalloids, avoiding hypovolemia and the use of colloids. Antiplatelet therapy (aspirin 100 mg) was not suspended perioperatively.

Perfusion imaging was part of the standard postoperative work-up. Non-contrast CT, CT angiography and CT perfusion were obtained within 24 h of surgery and repeated on postoperative day 7. In addition, MR angiography with NOVA study and digital subtraction angiography were performed in all cases during the first postoperative week to assess bypass patency and functionality. Any clinical change, such as a new or worsening neurological deficit, headache, altered consciousness, or seizure, prompted additional CT angiography, CT perfusion and/or MRI, according to clinical needs.

When hyperperfusion was detected, the main objective was to lower the target blood pressure range (systolic 100–120 mmHg), in line with the published literature, while maintaining close neurological and hemodynamic surveillance.

Prophylactic antiseizure medication was not routinely administered to patients without a history of seizures and with a normal preoperative EEG. Conversely, in patients affected by preoperative seizures or demonstrating EEG abnormalities, antiseizure medication was generally started. In case of postoperative seizures, benzodiazepines were administered as an acute first-line treatment followed, according to EEG findings and clinical needs, by long-term therapy (generally levetiracetam or phenytoin) through a multidisciplinary discussion.

### 2.3. Variables

The primary outcome variable was the occurrence of at least one epileptic seizure during the in-hospital postoperative period (Seizures in hospital: 0/1), defined as clinical manifestations of abnormal transient or repetitive motor movement, sensory and/or psychic phenomena with or without electroencephalographic (EEG) changes. For procedures complicated by postoperative seizures, available clinical records were additionally reviewed to characterize seizure semiology, clinical course, postoperative EEG findings, antiepileptic treatment, associated postoperative imaging abnormalities, and the temporal relationship between seizure onset and documented cerebral hyperperfusion. Postoperative EEG findings were classified according to the presence or absence of documented epileptiform abnormalities. Detailed characteristics of the postoperative seizure events are provided in Supplementary Table S1. The primary exposure of interest was cerebral hyperperfusion syndrome (Hyperperfusion: 0/1). Postoperative cerebral hyperperfusion was assessed using CT perfusion imaging and was defined as a regional increase in cerebral blood flow (CBF) and cerebral blood volume (CBV), accompanied by decreased mean transit time (MTT) and Tmax in the revascularized territory relative to the contralateral hemisphere. A side-to-side difference of approximately >20% was generally considered supportive of hyperperfusion; however, because of the retrospective nature of the study and the inherent variability of perfusion measurements, this threshold was not applied as an absolute diagnostic cutoff. Hyperperfusion was ultimately determined by the overall perfusion pattern and neuroradiological assessment. This approach is consistent with previously described CT-perfusion patterns of cerebral hyperperfusion, characterized by increased regional CBF and CBV with shortened transit-time parameters (as described by Lin et al. [13]). For cases presenting both hyperperfusion and postoperative seizures, the timing of documented hyperperfusion relative to seizure onset was additionally reviewed.

Covariates included demographic variables (age, sex, BMI), vascular risk factors (hypertension, diabetes mellitus, dyslipidemia, smoking), neurological history (prior epilepsy diagnosis, preoperative EEG seizure activity, antiepileptic drug use, psychiatric disorders, dysthyroidism), intraoperative hemodynamic measurements (STA flowmetry by Doppler), cerebrovascular imaging data (preoperative Suzuki angiographic grading, postoperative MR-NOVA cerebral blood flow), functional status (modified Rankin Scale, mRS).

All included procedures incorporated a direct STA–MCA bypass, either as direct revascularization alone or as part of combined direct–indirect revascularization. Procedures consisting exclusively of indirect revascularization, OA–MCA bypass, or double-barrel bypass were excluded. Detailed recipient-vessel territory, craniotomy characteristics, and extent of the indirect component were not systematically encoded throughout the retrospective study period and were therefore not included as analytical covariates.

### 2.4. Statistical Methods

Continuous variables are reported as median with interquartile range (IQR). Categorical variables are expressed as absolute frequencies and percentages. Comparisons between patients with and without postoperative seizures were performed using the Mann-Whitney U test for continuous variables and Fisher’s exact test for categorical variables, given the small sample sizes and non-normal distributions.

For regression analysis, we employed Firth’s penalized-likelihood logistic regression, implemented via an iterative reweighted least squares algorithm with a Jeffreys prior penalty. This method is specifically indicated for settings with small samples, rare outcomes, or risk of complete/quasi-complete separation—all of which apply to the present dataset. Firth regression yields finite, less biased parameter estimates compared to standard maximum likelihood logistic regression in such settings.

Univariate Firth models were first fitted for each covariate individually. Variables with a significant *p* value in univariate analysis, together with clinically relevant predictors identified a priori (history of epilepsy, antiepileptic treatment, preoperative EEG seizures), were included in the multivariable model. To limit model overfitting, three separate multivariable models were specified, each centered on one of the variables that was statistically significant in the univariate analysis. Results are reported as odds ratios (OR) with 95% confidence intervals (CI) derived from the Wald statistic on the penalized estimates. A two-sided *p* < 0.05 was considered statistically significant. All statistical analyses were conducted in Python 3.12 (Python Software Foundation, Wilmington, DE, USA) using custom implementations of Firth’s algorithm, with SciPy and stats models for supporting analyses. Visualizations were produced with matplotlib and seaborn.

Additional sensitivity analyses were performed to evaluate the robustness of the principal findings. Because some patients contributed more than one procedure, within-patient correlation was assessed using generalized estimating equations (GEE) with an exchangeable correlation structure and patient identifier as the clustering variable. Patient-level sensitivity analyses restricted to one procedure per patient were additionally performed. A combined multivariable Firth model including hyperperfusion, diabetes mellitus, hemispheric laterality, history of epilepsy, antiepileptic treatment, preoperative EEG seizure activity, and age was explored to assess the simultaneous contribution of the principal candidate variables. Given the limited number of outcome events relative to the number of covariates, this combined model was considered exploratory. Additional exploratory analyses evaluated the association between diabetes and hyperperfusion and the relationship between postoperative EEG abnormalities and hyperperfusion using Fisher’s exact test.

## 3. Results

### 3.1. Sample Characteristics and Outcome Distribution

The study included 75 hemispheric procedures. The median age was 45.0 years (IQR: 37.5–52.0). The cohort was predominantly female (84.0%, n = 63). Postoperative epileptic seizures occurred in 14 of 75 procedures (18.7%). Hyperperfusion syndrome was identified in 6 procedures (8.0%).

Among the 6 procedures complicated by hyperperfusion, 4 (66.7%) were associated with postoperative seizures, compared with 10 of the remaining 69 procedures without hyperperfusion (14.5%). Table 1 summarizes baseline characteristics stratified by seizure occurrence. Perioperative quantitative hemodynamic measurements did not differ significantly between patients with and without postoperative seizures. Neither intraoperative STA flowmetry (No Seizures 31.0 (25.5–48.0) vs. Seizures 20.0 (13.8–36.8), *p* value 0.104) nor MRI NOVA cerebral blood flow measurements (No Seizures 46.1 (32.2–79.5) vs. Seizures 82.0 (35.0–102.0), *p* value 0.487) were associated with seizure occurrence. Although patients who developed seizures tended to have lower STA flowmetry values and higher MR-NOVA cerebral blood flow measurements, these differences did not reach statistical significance.

**Figure 1:**
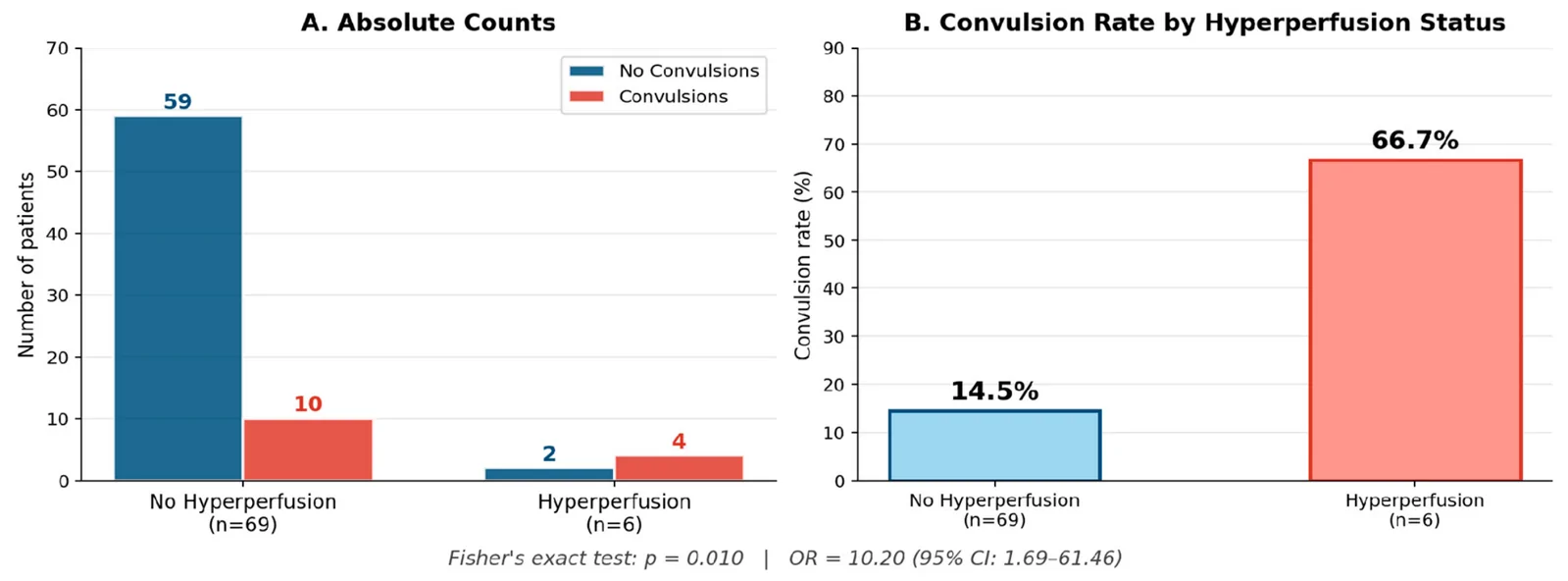
Postoperative seizure rates by hyperperfusion status. Left panel shows absolute counts; right panel shows proportional seizure rates. Fisher’s exact test *p* = 0.010; OR = 10.20 (95% CI: 1.69–61.46).

Notable differences between groups included a higher frequency of hyperperfusion in patients with seizures (28.6% vs. 3.3%, *p* = 0.010, Figure 1)), higher prevalence of diabetes mellitus (30.8% vs. 6.6%, *p* = 0.028), and a higher proportion of left-hemisphere procedures (78.6% vs. 44.3% in the seizure group vs. no-seizure group; right hemisphere: 21.4% vs. 55.7%, *p* = 0.036). Furthermore, Supplementary Figure S1 shows the comparative prevalence of key risk factors between the seizure and no-seizure groups. Here, hyperperfusion and diabetes were the two variables with statistically significant differences (* *p* < 0.05).

### 3.2. Univariate Firth Logistic Regression

**Figure 2:**
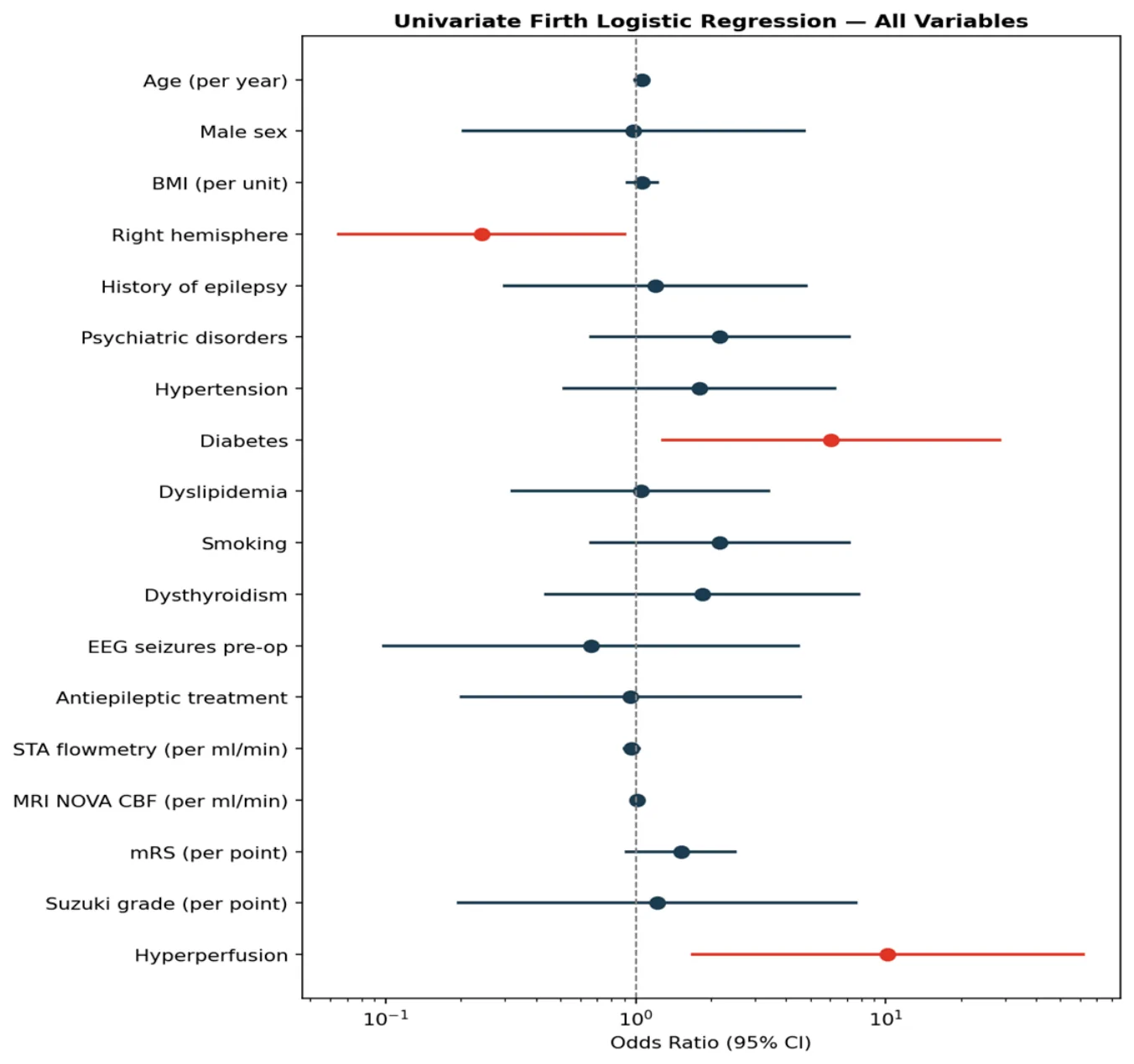
Forest plot of univariate Firth logistic regression. Red markers indicate statistically significant associations (*p* < 0.05). Horizontal lines represent 95% confidence intervals. Reference line at OR = 1.

In univariate Firth penalized logistic regression analyses, hyperperfusion syndrome demonstrated the strongest association with postoperative seizures (OR = 10.20, 95% CI: 1.69–61.46, *p* = 0.011). Diabetes mellitus was also a statistically significant predictor (OR = 6.05, 95% CI: 1.29–28.49, *p* = 0.023). Age showed a borderline association (OR = 1.06 per year, 95% CI: 0.99–1.12, *p* = 0.086). No other variables reached statistical significance in univariate analysis (Table 2 and Figure 2).

### 3.3. Multivariable Firth Logistic Regression

Three separate multivariable Firth logistic regression models were fitted on 74 complete observations, each incorporating one of the variables that reached statistical significance in univariate analysis (hyperperfusion, diabetes mellitus, or right hemisphere, respectively) together with history of epilepsy, antiepileptic treatment, and preoperative EEG seizures as a priori covariates, in order to limit overfitting given the limited number of outcome events. In Model A, hyperperfusion remained an independent predictor of postoperative seizures after adjustment (adjusted OR = 10.28, 95% CI: 1.64–64.35, *p* = 0.013). In Model B, diabetes mellitus remained independently associated with postoperative seizures (adjusted OR = 5.70, 95% CI: 1.17–27.77, *p* = 0.031). In Model C, right hemisphere did not reach statistical significance after adjustment (adjusted OR = 0.29, 95% CI: 0.08–1.04, *p* = 0.057). History of epilepsy, antiepileptic treatment, and preoperative EEG seizures were not associated with postoperative seizures in any of the three models (Table 3). Because hyperperfusion and diabetes could not be reliably evaluated within a single model given the limited number of outcome events, these findings should be interpreted as complementary rather than fully independent predictors; an exploratory combined model including both variables simultaneously is presented in Section 3.6.

### 3.4. Distribution of Clinical Variables and Risk Factors

**Figure 3:**
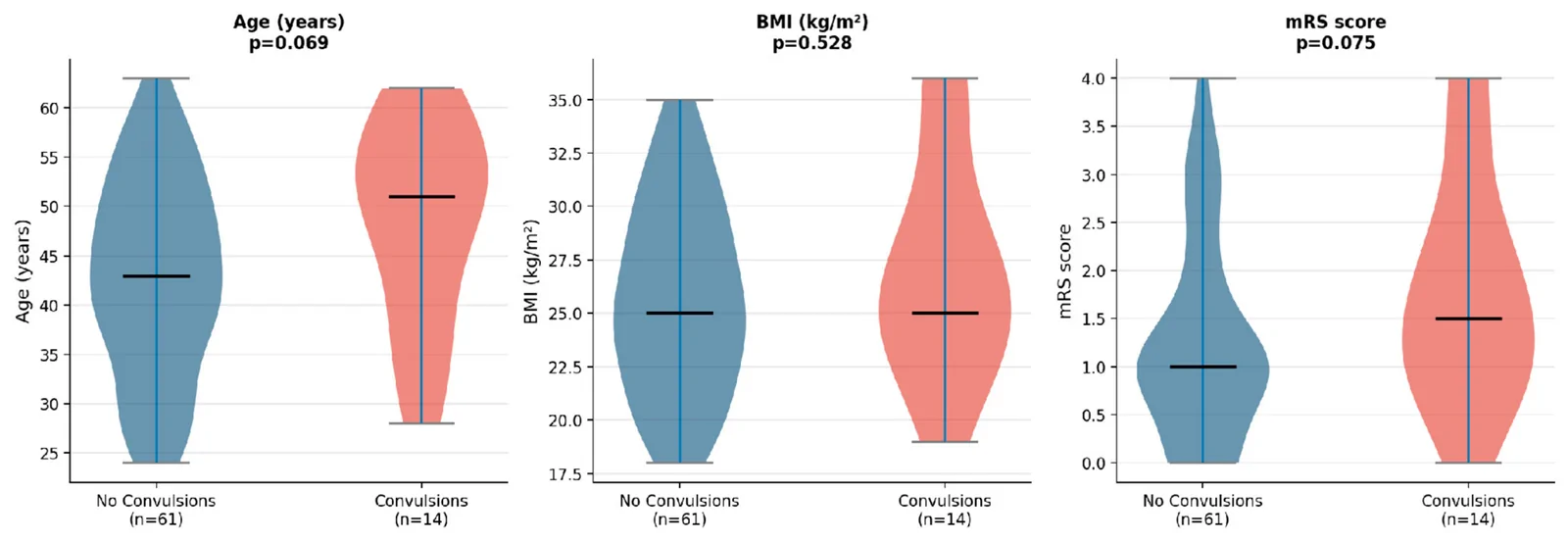
Cinical variables by postoperative convulsion status. Violin plots showing the distribution of age, BMI, and mRS by postoperative seizure status. Black horizontal lines represent medians. Mann-Whitney U *p*-values are shown for each comparison.

Figure 3 displays the distribution of continuous variables (age, BMI, mRS) stratified by postoperative seizure occurrence using violin plots. Age showed a trend toward older values in the seizure group (median 51.0 vs. 43.0 years, *p* = 0.069). BMI was similar between groups. mRS scores showed a borderline difference (*p* = 0.075), suggesting higher baseline disability in patients who experienced seizures.

### 3.5. Clinical and Electroencephalographic Characteristics of Postoperative Seizures

Among the 14 procedures complicated by postoperative seizures, most events were focal and transient, with clinical manifestations including expressive language disturbance, sensory or sensorimotor phenomena, speech arrest, ocular deviation, and clonic motor activity; a smaller number presented with generalized or focal-to-bilateral tonic-clonic seizures. Two events followed a persistent clinical course requiring intubation. Postoperative EEG information regarding epileptiform abnormalities was available in 13 cases. Four demonstrated epileptiform abnormalities, whereas nine had no documented epileptiform correlate. Both persistent events requiring intubation showed EEG abnormalities. Detailed clinical characteristics of the 14 seizure events are provided in Supplementary Table S1.

None of the four seizure cases with epileptiform EEG abnormalities had documented hyperperfusion, whereas hyperperfusion was present in four of nine seizure cases without epileptiform EEG abnormalities; this difference was not statistically significant (Fisher’s exact test, *p* = 0.228). Four procedures presented both postoperative hyperperfusion and seizures. Hyperperfusion was documented either on the same day as or before seizure onset in all four cases, with intervals of 0, 1, 2, and 1 days, respectively. No seizure preceded the identification of hyperperfusion. Among the seizure cases for which detailed postoperative imaging information could be retrieved, three had documented new ischemic lesions, two had subdural hematomas, and one had pneumocephalus. These findings were considered descriptive and were not incorporated into the regression models.

### 3.6. Exploratory and Sensitivity Analyses

Hyperperfusion occurred in 2 procedures in patients with diabetes (25.0%) and in 4 procedures in patients without diabetes (6.1%); this difference did not reach statistical significance (Fisher’s exact test, *p* = 0.124). Among procedures without documented hyperperfusion, postoperative seizures occurred in 2 procedures in patients with diabetes (33.3%) compared with 7 procedures in patients without diabetes (11.3%). The association between hyperperfusion and postoperative seizures remained consistent in sensitivity analyses accounting for within-patient clustering. The procedure-level Firth analysis yielded an OR of 10.20 (*p* = 0.011), while the patient-clustered GEE analysis yielded an OR of 15.96 (*p* = 0.005). Patient-level analyses restricted to one procedure per patient similarly demonstrated an association between hyperperfusion and postoperative seizures. Diabetes also remained associated with postoperative seizures in the GEE analysis (OR 7.11, *p* = 0.006), although results were less consistent across the patient-level sensitivity analyses. In contrast, the association with hemispheric laterality was not consistently maintained. In an exploratory combined Firth model including hyperperfusion, diabetes, hemispheric laterality, history of epilepsy, antiepileptic treatment, preoperative EEG seizure activity, and age, hyperperfusion showed an OR of 6.01 (95% CI 0.81–44.54; *p* = 0.079) and diabetes an OR of 3.52 (95% CI 0.61–20.18; *p* = 0.158). Neither retained conventional statistical significance when simultaneously included in this highly parameterized model. Complete results of the sensitivity analyses are presented in Supplementary Table S2.

## 4. Discussion

In this retrospective cohort of adult patients undergoing STA–MCA revascularization for Moyamoya angiopathy, early postoperative seizures occurred in approximately 19% of procedures. Postoperative cerebral hyperperfusion showed the strongest and most consistent association with early postoperative seizures across the primary and sensitivity analyses. Diabetes mellitus also showed an association with postoperative seizures, although this finding was less consistent across sensitivity analyses. In contrast, hemispheric laterality was not consistently associated after adjustment and patient-level sensitivity analyses. Pre-existing epilepsy, preoperative EEG abnormalities, and chronic antiepileptic treatment were not associated with postoperative seizure occurrence.

The incidence of postoperative seizures reported in the literature varies considerably. Jin et al. reported an overall seizure rate of 18.9%, distinguishing between early and delayed postoperative events, whereas Bao et al. described a substantially lower incidence of 3.19% [8,14]. Such variability is likely explained by differences in patient selection, surgical strategy (direct, indirect, or combined revascularization), duration of follow-up, and, importantly, the definition of postoperative seizures. Studies focusing exclusively on seizures occurring in the context of cerebral hyperperfusion syndrome consistently report lower rates. Accordingly, the meta-analysis by Yu et al. found a pooled incidence of hyperperfusion-related seizures of 5.3%, while the recent proportional meta-analysis by Luck et al. reported an overall postoperative seizure rate of 4.61% together with a pooled hyperperfusion syndrome incidence of 14.96% [15,16]. Our higher incidence probably reflects the inclusion of all clinically detected early postoperative seizures during the in-hospital period rather than only seizures fulfilling predefined hyperperfusion criteria. The temporal analysis in the present cohort provides additional support for this association. In all four procedures presenting both hyperperfusion and seizures, hyperperfusion was identified either before or on the same day as seizure onset, and no seizure preceded its identification. Although this temporal sequence cannot establish causality in a retrospective study, it reduces the possibility that the observed association merely reflects hyperperfusion diagnosed as a consequence of seizure-related imaging.

Our findings reinforce accumulating evidence supporting cerebral hyperperfusion as an important mechanism underlying postoperative seizures after direct revascularization. Huang et al. identified postoperative hyperperfusion among the principal predictors of postoperative epilepsy in adult Moyamoya patients, while Liu et al. similarly observed an association between hyperperfusion syndrome and postoperative epilepsy following combined revascularization [12,17]. Although these studies differed in surgical strategy and outcome definition, they consistently suggest that postoperative hemodynamic disturbances play a central role in seizure development.

In contrast, Niu et al. reported that postoperative seizures were primarily associated with cerebral infarction rather than hyperperfusion, with no seizures occurring among patients who developed hyperperfusion syndrome [11]. Rather than representing conflicting evidence, these observations likely reflect different biological mechanisms leading to postoperative seizures. One pathway may be driven by structural cortical injury secondary to perioperative ischemia or infarction, whereas another appears to result from excessive reperfusion of chronically hypoperfused cortex with impaired autoregulatory capacity.

The biological basis of hyperperfusion-related seizures is well supported by previous experimental and clinical observations. Chronic cerebral hypoperfusion impairs cerebrovascular autoregulation, rendering cortical tissue vulnerable to abrupt increases in cerebral blood flow after direct bypass. The resulting excessive regional perfusion may promote vasogenic edema, disruption of the blood-brain barrier, oxidative stress, and cortical neuronal hyperexcitability, thereby lowering the seizure threshold [10,18]. Wang et al. further demonstrated, using intraoperative perfusion-weighted magnetic resonance imaging, that marked increases in regional cerebral blood flow immediately after bypass are associated with subsequent development of hyperperfusion syndrome, providing imaging evidence supporting this pathophysiological model [9].

Interestingly, factors traditionally considered markers of epileptogenic susceptibility did not appear to influence early postoperative seizure risk in our cohort. Neither a previous history of epilepsy, preoperative EEG abnormalities, nor chronic antiepileptic treatment demonstrated an association with postoperative seizures. This observation is consistent with the findings of Niu et al. [11], although Liu et al. reported an association between preoperative epilepsy and postoperative seizures [17]. Because detailed information regarding antiepileptic drug dosage, serum drug concentrations, longitudinal seizure control, and serial preoperative EEG examinations was not systematically available in our cohort, the present data cannot determine whether the absence of an association reflects effective preoperative seizure control. At our institution, patients with a history of epilepsy undergo routine preoperative neurological evaluation, including EEG when clinically indicated, and antiepileptic therapy is optimized before surgery whenever epileptiform activity or inadequate seizure control is identified. Previous studies have demonstrated that repeated EEG examinations can increase the detection of epileptiform abnormalities [19]. However, evidence that serial preoperative EEG examinations reduce postoperative seizure risk following Moyamoya revascularization remains insufficient. Therefore, although EEG may contribute to the preoperative assessment of patients with known or suspected epilepsy, our findings do not support conclusions regarding the preventive efficacy of serial EEG monitoring or preoperative antiepileptic treatment optimization.

Another noteworthy finding was the higher prevalence of diabetes mellitus among patients who developed postoperative seizures. This observation should be considered hypothesis-generating rather than confirmatory. Diabetes mellitus is characterized by endothelial dysfunction, microvascular injury, oxidative stress, and blood–brain barrier impairment, all of which have been associated with increased neuronal excitability and seizure susceptibility. These mechanisms may further compromise the already impaired cerebrovascular autoregulation characteristic of Moyamoya angiopathy and increase vulnerability to reperfusion-related neurological injury [20,21]. Experimental and clinical studies have demonstrated that chronic hyperglycemia disrupts blood–brain barrier integrity and increases its permeability through endothelial injury and loss of tight-junction proteins [22,23]. In addition, diabetes promotes persistent neuroinflammatory responses characterized by activation of astrocytes and microglia and increased production of pro-inflammatory cytokines, mechanisms that enhance neuronal hyperexcitability and lower the seizure threshold [21,24]. In our cohort, hyperperfusion was numerically more frequent among patients with diabetes, this difference did not reach statistical significance. Moreover, postoperative seizures also occurred among diabetic patients without documented hyperperfusion. Although these mechanisms remain speculative and only represent biological hypotheses rather than a demonstrated pathophysiological pathway in the context of Moyamoya surgery and the presence of postoperative epilepsy, our findings raise the possibility that diabetes may increase susceptibility to postoperative hemodynamic injury and deserve further evaluation in larger cohorts.

Left-sided procedures were associated with postoperative seizures in the univariate analysis but lost significance after adjustment for potential confounders. As described by Fisher et al., focal seizures involving the language-dominant hemisphere commonly manifest with aphasia or other language disturbances, symptoms that are readily recognized during postoperative neurological examination [25]. Consequently, seizures affecting the dominant hemisphere may be more frequently detected than those arising from the non-dominant hemisphere, introducing a potential ascertainment bias. Nevertheless, given the limited sample size and the loss of significance after adjustment (also in the patient-level sensitivity analyses) this finding should be regarded as exploratory.

## 5. Clinical Implications

The identification of postoperative hyperperfusion as the principal factor associated with early seizures has several practical implications. First, patients demonstrating clinical or radiological evidence of hyperperfusion may benefit from closer neurological surveillance during the early postoperative period, including EEG-monitoring when clinically indicated. Second, meticulous postoperative blood pressure management, already recommended to reduce hyperperfusion-related hemorrhagic complications, may also contribute to reducing seizure risk, although prospective evidence remains limited. Finally, routine postoperative assessment of cerebral perfusion using transcranial Doppler, MR, or CT perfusion may facilitate early identification of patients at greatest risk and allow timely implementation of individualized postoperative management strategies. Whether prophylactic antiepileptic treatment should be routinely considered in patients with confirmed hyperperfusion remains uncertain and warrants prospective investigation. Although requiring external validation, diabetic patients may represent a subgroup warranting particularly careful postoperative hemodynamic monitoring after direct revascularization.

## 6. Limitations

First, the retrospective, single-center design limits causal inference and generalizability. Second, the number of postoperative seizure and hyperperfusion events was relatively small, resulting in wide confidence intervals and limiting the number of covariates that could be simultaneously incorporated into multivariable models. The combined multivariable analysis should therefore be regarded as exploratory. Third, some patients contributed more than one procedure. Although sensitivity analyses accounting for within-patient clustering supported the principal association with hyperperfusion, residual patient-level dependence cannot be completely excluded. Fourth, detailed operative characteristics, including recipient artery territory, craniotomy characteristics, and extent of the indirect revascularization component, as well as standardized patient-level postoperative blood pressure and fluid-management parameters, were not systematically encoded across the 18-year study period and could not be reliably incorporated as potential confounders. Fifth, detailed postoperative imaging variables such as new ischemic lesions and hemorrhagic complications were not systematically available for the complete cohort. Their potential contribution to postoperative seizure development therefore could not be formally adjusted for. Similarly, detailed antiepileptic drug dosages, serum concentrations, longitudinal seizure control, and serial EEG data were unavailable. Finally, intraoperative STA flowmetry and MR-NOVA cerebral blood flow measurements were available only in subsets of procedures, substantially reducing statistical power to detect associations with postoperative seizures.

## 7. Conclusions

Early postoperative seizures after STA–MCA bypass in adult Moyamoya angiopathy were most consistently associated with postoperative cerebral hyperperfusion across the primary and sensitivity analyses. Diabetes mellitus emerged as a potential clinical susceptibility factor, although this association requires validation in larger cohorts. These findings support a relevant role of acute postoperative hemodynamic disturbances in seizure development following cerebral revascularization. Early recognition of hyperperfusion may facilitate risk-stratified postoperative surveillance and individualized management.

## Figures and Tables

**Table 1:** Baseline Characteristics Stratified by Postoperative Seizure Status.

Variable	Total (n = 75)	No Seizures (n = 61)	Seizures (n = 14)	*p*-Value
Age, years	45.0 (37.5–52.0)	43.0 (37.0–50.0)	51.0 (40.8–55.5)	0.069
Sex, Male	12/75 (16.0%)	10/61 (16.4%)	2/14 (14.3%)	1
BMI, kg/m^2^	25.0 (23.0–29.0)	25.0 (22.5–29.0)	25.0 (24.0–29.0)	0.528
Right hemisphere	37/75 (49.3%)	34/61 (55.7%)	3/14 (21.4%)	0.036 *
History of epilepsy	16/74 (21.6%)	13/61 (21.3%)	3/13 (23.1%)	1
Psychiatric disorders	28/74 (37.8%)	21/61 (34.4%)	7/13 (53.8%)	0.219
Hypertension	42/74 (56.8%)	33/61 (54.1%)	9/13 (69.2%)	0.371
Diabetes	8/74 (10.8%)	4/61 (6.6%)	4/13 (30.8%)	0.028 *
Dyslipidemia	39/74 (52.7%)	32/61 (52.5%)	7/13 (53.8%)	1
Smoking	28/74 (37.8%)	21/61 (34.4%)	7/13 (53.8%)	0.219
Dysthyroidism	10/72 (13.9%)	7/59 (11.9%)	3/13 (23.1%)	0.373
EEG seizures before surgery	10/74 (13.5%)	9/61 (14.8%)	1/13 (7.7%)	0.68
Antiepileptic treatment	11/72 (15.3%)	9/59 (15.3%)	2/13 (15.4%)	1
STA flowmetry (mL/min)	28.0 (21.0–48.0)	31.0 (25.5–48.0)	20.0 (13.8–36.8)	0.104
MRI NOVA (mL/min, median IQR)	46.2 (33.5–94.2)	46.1 (32.2–79.5)	82.0 (35.0–102.0)	0.487
Hyperperfusion (%)	6/75 (8.0%)	2/61 (3.3%)	4/14 (28.6%)	0.010*
mRS (median IQR)	1.0 (1.0–2.0)	1.0 (1.0–2.0)	1.5 (1.0–2.0)	0.075

Continuous variables: median (IQR). Categorical variables: n (%). *p*-values from Mann-Whitney U (continuous) or Fisher’s exact test (categorical). * *p* < 0.05. Abbreviations: BMI, body mass index; STA, superficial temporal artery; EEG, electroencephalogram; mRS, modified Rankin Scale; IQR, interquartile range.

**Table 2:** Univariate Firth Penalized Logistic Regression Results.

Variable	n	OR	95% CI Lower	95% CI Upper	*p*-Value
Age (per year)	75	1.06	0.99	1.12	0.086
Male sex	75	0.98	0.21	4.69	0.981
BMI (per unit)	70	1.06	0.93	1.22	0.389
Right hemisphere	75	0.24	0.07	0.90	0.035 *
History of epilepsy	74	1.20	0.30	4.79	0.799
Psychiatric disorders	74	2.17	0.66	7.15	0.201
Hypertension	74	1.80	0.52	6.24	0.357
Diabetes	74	6.05	1.29	28.49	0.023 *
Dyslipidemia	74	1.05	0.32	3.40	0.939
Smoking	74	2.17	0.66	7.15	0.201
Dysthyroidism	74	1.84	0.44	7.77	0.406
EEG seizures pre-op	74	0.66	0.10	4.48	0.673
Antiepileptic treatment	74	0.95	0.20	4.54	0.953
STA flowmetry (per mL/min)	25	0.96	0.90	1.03	0.250
MRI NOVA CBF (per mL/min)	36	1.01	0.99	1.03	0.386
mRS (per point)	74	1.52	0.92	2.50	0.102
Suzuki grade (per point)	20	1.22	0.20	7.59	0.833
Hyperperfusion	75	10.20	1.69	61.46	0.011 *

OR: odds ratio; CI: confidence interval. Firth’s penalized-likelihood estimates. * *p* < 0.05.

**Table 3:** Multivariable Firth Penalized Logistic Regression Results.

Variable	Adjusted OR	95% CI Lower	95% CI Upper	*p*-Value
**Model A**				
Hyperperfusion	10.28	1.64	64.35	0.013 *
History of epilepsy	0.95	0.13	6.76	0.959
No antiepileptic treatment	0.81	0.09	7.57	0.856
EEG seizures pre-op	0.55	0.07	4.62	0.585
**Model B**				
Diabetes	5.70	1.17	27.77	0.031 *
History of epilepsy	1.77	0.28	11.24	0.545
No antiepileptic treatment	1.37	0.15	12.27	0.776
EEG seizures pre-op	0.99	0.13	7.57	0.996
**Model C**				
Right hemisphere	0.29	0.08	1.04	0.057
History of epilepsy	1.16	0.19	7.01	0.871
No antiepileptic treatment	0.84	0.10	7.26	0.875
EEG seizures pre-op	0.71	0.09	5.57	0.745

Adjusted OR: odds ratio adjusted for all variables in the model; CI: confidence interval. Firth’s penalized-likelihood estimates. n = 74. * *p* < 0.05.

## Data Availability

Due to hospital regulations, the dataset cannot be made publicly accessible. However, cohort-level data may be provided by the corresponding author upon reasonable request.

## Supplementary Materials

The following supporting information can be downloaded at: https://www.mdpi.com/article/10.3390/jcm15218448/s1, Table S1. Clinical, electroencephalographic, and management characteristics of postoperative seizure cases: Abbreviations: EEG, electroencephalography; LVT, levetiracetam; ND, no data. “/” indicates no alteration or not applicable to that case, according to the source dataset. Table S2. Additional exploratory and sensitivity analyses: Abbreviations: CI, confidence interval; EEG, electroencephalography; GEE, generalized estimating equation; OR, odds ratio. Analyses are sensitivity/exploratory analyses and should not be interpreted as replacing the prespecified primary models. Figure S1. Prevalence of comorbidities and clinical risk factors by postoperative seizure status. Asterisks (*) denote statistically significant differences (Fisher’s exact test *p* < 0.05): Hyperperfusion (*p* = 0.010) and Diabetes (*p* = 0.028).
