# Supplementary material for: Predictors of Early Postoperative Seizures Following STA–MCA Bypass in Adult Moyamoya Angiopathy
Source: J Clin Med. 2026 Oct 30;15(21):8448. doi: 10.3390/jcm15218448 (PMC13763693; doi:10.3390/jcm15218448)
Supplement: Supplementary file 1 [file jcm-15-08448-s001.zip › jcm-4507507-supplementary.pdf]

Supplementary Tables and Figure

Supplementary Table S1. Clinical, electroencephalographic, and management characteristics of postoperative seizure cases

Case	History of epilepsy	Preoperative EEG seizure activity	Preoperative antiseizure treatment	Hyperperfusion-to-seizure interval (days)	Seizure type	Clinical characteristics	Clinical course / duration	Postoperative EEG epileptiform abnormalities	Management	Other management
1	No	No	No	0	Focal	Expressive language disturbance and sensory symptoms (paresthesia)	Transient; spontaneous resolution within minutes	No	LVT	EEG hypercapnia and polysomnography
2	No	No	No	/	Focal	Expressive language disturbance	Transient; spontaneous resolution within minutes	Yes	Lamictal	EEG
3	Yes	No	Yes	1	Focal	Motor seizure (eye deviation, orobuccal movements, and clonic upper-limb movements)	Transient; resolution with medication within minutes	No	LVT increased + Vimpat	EEG
4	No	No	No	/	Focal and generalized	Tonic-clonic seizure with loss of consciousness	Persistent; required intubation	Yes	Phenytoin	EEG
5	Yes	No	Yes	/	Focal	Sensory and motor symptoms	Transient; spontaneous resolution	No	LVT increased + Frisium	EEG
6	No	No	No	/	Focal	Speech arrest and absence-like episode	Transient; spontaneous resolution	No	LVT increased	/

7	No	No	No	/	Generalized	Tonic-clonic seizure	Persistent; required intubation	Yes	Phenytoin	Intubation and EEG
8	No	No	No	/	Focal	Language disturbance	Transient; spontaneous resolution	No	LVT	/
9	ND	ND	ND	/	Generalized	Tonic-clonic seizure	Transient	No	LVT increased	/
10	Yes	No	No	2	Focal	Expressive language disturbance	Transient	No	LVT	EEG
11	No	Yes	No	/	Focal	Expressive language disturbance	Transient; spontaneous resolution	Yes	LVT	EEG
12	No	No	No	/	Focal	Expressive language disturbance	Transient; spontaneous resolution	No	ND	/
13	No	Yes	No	/	Focal	Language disturbance	ND	No	ND	/
14	No	No	No	1	Focal and generalized	Focal motor to tonic-clonic	Transient; spontaneous resolution	No	LVT	EEG

Abbreviations: EEG, electroencephalography; LVT, levetiracetam; ND, no data. "/" indicates no alteration or not applicable to that case, according to the source dataset.

**Supplementary Table S2. Additional exploratory and sensitivity analyses**				
**Analysis**	**Exposure / comparison**	**Estimate**	**p value**	**Interpretation / note**
Primary procedure-level Firth analysis	Hyperperfusion → postoperative seizure	OR 10.20	0.011	Primary procedure-level association reported in the manuscript.
Patient-clustered GEE sensitivity analysis	Hyperperfusion → postoperative seizure	OR 15.96	0.005	Accounts for within-patient clustering.
Patient-level sensitivity analysis 1	Hyperperfusion → postoperative seizure	OR 44.00	0.004	One-procedure-per-patient sensitivity approach.

Patient-level sensitivity analysis 2	Hyperperfusion → postoperative seizure	OR 19.00	0.009	Alternative one-procedure-per-patient sensitivity approach.
Patient-clustered GEE sensitivity analysis	Diabetes mellitus → postoperative seizure	OR 7.11	0.006	Association maintained after accounting for clustering.
Patient-level sensitivity analysis 1	Diabetes mellitus → postoperative seizure	OR 10.57	0.017	Association present in this patient-level sensitivity approach.
Patient-level sensitivity analysis 2	Diabetes mellitus → postoperative seizure	OR 5.12	0.141	Association did not reach statistical significance in this sensitivity approach.
Exploratory combined Firth model	Hyperperfusion → postoperative seizure	OR 6.01	0.079	Model included hyperperfusion, diabetes, laterality, history of epilepsy, antiseizure treatment, preoperative EEG seizure activity, and age; 75 complete observations and 14 seizure events.
Exploratory combined Firth model	Diabetes mellitus → postoperative seizure	OR 3.52	0.158	Same combined model; association did not retain conventional statistical significance.
Exploratory Fisher exact test	Diabetes mellitus vs. hyperperfusion	25.0% (2/8) vs. 6.1% (4/66)	0.124	Hyperperfusion was numerically more frequent in procedures involving patients with diabetes, without statistical significance.
Exploratory Fisher exact test	Postoperative EEG epileptiform abnormalities vs. hyperperfusion among seizure cases	0/4 vs. 4/9	0.228	No hyperperfusion among EEG-positive seizure cases versus hyperperfusion in 4/9 cases without documented epileptiform EEG abnormalities.

Abbreviations: CI, confidence interval; EEG, electroencephalography; GEE, generalized estimating equation; OR, odds ratio. Analyses are sensitivity/exploratory analyses and should not be interpreted as replacing the prespecified primary models.

Figure S1: Prevalence of Risk Factors by postoperative seizure status.

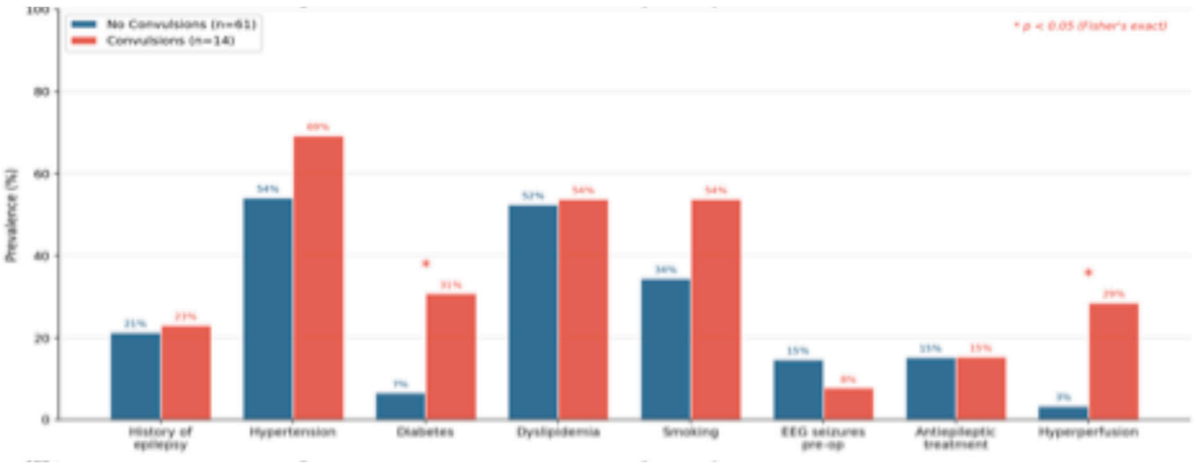

Supplementary Figure 1. Prevalence of comorbidities and clinical risk factors by postoperative seizure status. Asterisks (*) denote statistically significant differences (Fisher's exact test p<0.05): Hyperperfusion (p = 0.010) and Diabetes (p = 0.028).
